# The dependence of neuronal encoding efficiency on Hebbian plasticity and homeostatic regulation of neurotransmitter release

**DOI:** 10.3389/fncel.2015.00164

**Published:** 2015-04-28

**Authors:** Faramarz Faghihi, Ahmed A. Moustafa

**Affiliations:** ^1^Center for Neural Informatics, Structures, and Plasticity, Krasnow Institute for Advanced Study, George Mason UniversityFairfax, VA, USA; ^2^Department of Veterans Affairs, New Jersey Health Care SystemEast Orange, NJ, USA; ^3^School of Social Sciences and Psychology and Marcs Institute for Brain and Behavior, University of Western SydneySydney, NSW, Australia

**Keywords:** retrograde messenger, neurotransmitter release, homeostatic regulation, neural communication, Hebbian mechanism

## Abstract

Synapses act as information filters by different molecular mechanisms including retrograde messenger that affect neuronal spiking activity. One of the well-known effects of retrograde messenger in presynaptic neurons is a change of the probability of neurotransmitter release. Hebbian learning describe a strengthening of a synapse between a presynaptic input onto a postsynaptic neuron when both pre- and postsynaptic neurons are coactive. In this work, a theory of homeostatic regulation of neurotransmitter release by retrograde messenger and Hebbian plasticity in neuronal encoding is presented. Encoding efficiency was measured for different synaptic conditions. In order to gain high encoding efficiency, the spiking pattern of a neuron should be dependent on the intensity of the input and show low levels of noise. In this work, we represent spiking trains as zeros and ones (corresponding to non-spike or spike in a time bin, respectively) as words with length equal to three. Then the frequency of each word (here eight words) is measured using spiking trains. These frequencies are used to measure neuronal efficiency in different conditions and for different parameter values. Results show that neurons that have synapses acting as band-pass filters show the highest efficiency to encode their input when both Hebbian mechanism and homeostatic regulation of neurotransmitter release exist in synapses. Specifically, the integration of homeostatic regulation of feedback inhibition with Hebbian mechanism and homeostatic regulation of neurotransmitter release in the synapses leads to even higher efficiency when high stimulus intensity is presented to the neurons. However, neurons with synapses acting as high-pass filters show no remarkable increase in encoding efficiency for all simulated synaptic plasticity mechanisms. This study demonstrates the importance of cooperation of Hebbian mechanism with regulation of neurotransmitter release induced by rapid diffused retrograde messenger in neurons with synapses as low and band-pass filters to obtain high encoding efficiency in different environmental and physiological conditions.

## Introduction

Neurons as the computational engines of the brain communicate with other neurons via synapses as conveyers of information. Neuronal firing and synaptic transmission between neurons form the building blocks for coding, processing, and storage of information in the brain (Salinas and Sejnowski, 2001). The spiking of a neuron in response to a stimulation by inputs is expected to be non-random (random means spontaneous spiking or noise) and to be dependent on input intensity. Therefore, the spiking pattern of a neuron conveys high levels of information about its inputs (high encoding efficiency) when the noise in the spiking pattern is minimized while the variation in spiking is maximized. On the other hand, low encoding efficiency is gained when a spiking pattern show low variability or high levels of noise (van Steveninck et al., 1997; Onken et al., 2014). Generally speaking, neural encoding efficiency is decreased when firing rate of a neural response is either high or very low which leads to a low variability in neural response. A low variation in a spiking pattern causes a low efficiency of encoding of either different stimuli or different intensity of a given stimulus presented to the neural system. Moreover, the diversity of neural spiking strongly depends on the properties of their synapses which remarkably vary in different types of neurons. This quantity is usually calculated using “mutual information” between a spike train and the stimulus as an information theoretic approach (Kumbhani et al., 2007; Faghihi et al., 2013; Fan, 2014; Jung et al., 2014). As neural systems should be able to detect a fluctuation in a stimulus intensity, a new encoding efficiency measure has been recently introduced which uses the geometric distance between stimulus and the response of a given neuron (Faghihi and Moustafa, 2015).

The increased complexity of synaptic protein networks was recently put forward as a potential correlate of mammalian cognitive abilities (Bayés et al., 2012; Nithianantharajah and Hannan, 2013). The diversity of synaptic plasticity mechanisms and their induced operation timescales suggest that synapses have complicated roles in information processing (Citri and Malenka, 2008; Lee et al., 2014; Yates, 2014). A long-term change in a synaptic structure provides a physiological substrate for learning and memory, whereas short-term changes support synaptic computations (Ziegler et al., 2015). The effect of an action potential transmitted from one neuron to another depends on the history of neural activity at either or both sides of the synapses such that their effect can last from milliseconds to months (Tetzlaff et al., 2012).

The release of a neurotransmitter as the main information transfer between neurons is a highly regulated process (Benfenati, 2007; Davis and Müller, 2014). Recently, neuroscience research has focused on the mechanisms of neurotransmitter release and their role in information encoding by neurons and neural network activity (Hardingham et al., 2013; Lazarevic et al., 2013; Kaeser and Regehr, 2014). Moreover, neurotransmitter release is not assured in response to synaptic stimulation, meaning that the process of neurotransmitter release in response to an action potential is essentially probabilistic.

Synapses are considered as filters that selectively and unreliably filter the flow of information between pre-and postsynaptic sites. Different synapses can show a variation in the initial probability of neurotransmitter release. Initial probability implies that the release probability may change over time. Regarding the concept of filtering, synapses are divided to three classes. For the majority of synapses in the central nervous system, the release probability at a defined synaptic contact is below 0.3, referred to as “reliably unreliable” release mechanism (Goda and Südhof, 1997). This kind of synapse is called a “high-pass filter” which is found for example in parallel fiber synapses. The synapses with high initial probability of neurotransmitter release, such as climbing fiber synapses, are called “low-pass filters” (Silver et al., 2003; Foster and Regehr, 2004; Murphy et al., 2004). Synapses with an intermediate release probability for example Schaffer collateral synapses are called “band-pass filters” (Abbott and Regehr, 2004; Rose et al., 2013).

Moreover, release probability is highly a dynamic process; it incorporates several forms of short-term plasticity mechanisms. The efficacy of synaptic transmission is dependent on the pattern of synaptic activation and the overall activity level of single neurons in a neural network. Activity-dependent changes in synaptic transmission arise from a large number of mechanisms known as synaptic plasticity (Abbott and Nelson, 2000; Lewis, 2014; Takeuchi et al., 2014; Welberg, 2014).

Functional synaptic plasticity includes homeostatic feedback mechanisms which enable neurons to respond to prolonged alterations in neuronal activity by regulating cellular excitability (Davis, 2006).

Investigating the complexity of homeostatic regulation of single neurons and neural circuits is thus fundamental for understanding brain function. Homeostatic signaling systems are thought to stabilize neural function through the regulation of ion channel density, neurotransmitter receptor abundance, and presynaptic neurotransmitter release (Davis, 2006, 2013; Marder and Goaillard, 2006; Bergquist et al., 2010; Thalhammer and Cingolani, 2014).

Homeostatic plasticity mechanisms are employed by neurons to alter membrane excitability and synaptic strength to adapt to changes in network activity. A number of cellular and molecular mechanisms have been identified as regulators of homeostatic plasticity (Maffei et al., 2012). Intrinsic membrane properties (intrinsic plasticity) as non-synaptic factors directly affect the probability that a neuron will spike in response to excitatory synaptic inputs (Kourrich et al., 2015). Based on the information of the underlying cellular mechanisms, neuronal homeostasis is categorized as the homeostatic control of intrinsic excitability of neurons by a change in ion channel expression (Turrigiano, 2011), synaptic efficacy, presynaptic neurotransmitter release, and network activity through regulation of inhibitory synapses (Turrigiano and Nelson, 2000, 2004; Davis, 2013).

Both excitatory and inhibitory synapses are subject to homeostatic regulation, and the form of plasticity present at a particular synapse likely depends on its function within a neuronal circuit. Feed-back inhibition and feed-forward inhibition as neural mechanism at network level may contribute to controlling input-output relationships in all parts of the brains (Tepper et al., 2008; Wang et al., 2013; Brown et al., 2014; Roux and Buzsáki, 2015) such that an impairment in their functionality may be associated with some mental disorders (Phillips and Uhlhaas, 2015; Ruddock et al., 2015). An important aspect of homeostatic plasticity is the dynamic interaction between excitatory and inhibitory inputs during homeostatic adaptation, as most of the studies to date have focused on either excitatory or inhibitory synapses individually. Therefore, it is highly important to study the inhibitory role of neurons in encoding efficiency of single neurons and neural populations as well.

Hebbian plasticity and homeostatic plasticity are the two major forms of activity-dependent plasticity that modify neuronal circuits (Turrigiano, 2008). Hebbian plasticity refers to plasticity that depends on the correlations between pre- and postsynaptic activity such that excitatory synapses that effectively drive a postsynaptic cell grow stronger. This is a positive feedback process that leads to synaptic instability in the absence of additional biological constraints (Turrigiano, 2008; Vitureira and Goda, 2013; Lee et al., 2014). Homeostatic plasticity is a negative feedback mechanism that typically involves non-specific scaling of all excitatory or inhibitory synapses onto a cell to oppose changes in overall activity levels. This is thought to maintain activity levels within a dynamic range and, more generally, to stabilize neuronal circuit function despite the positive feedback of Hebbian plasticity (Turrigiano, 2008). It is believed that homeostatic plasticity operates as a compensatory, negative feedback mechanism to maintain network stability (Turrigiano, 2008; Pozo and Goda, 2010). However, it is not fully known how these two forms of plasticity interact in biological systems (Shepherd and Huganir, 2007; Turrigiano, 2008, 2011; Vitureira and Goda, 2013). In models that combine Hebbian plasticity with homeostatic plasticity, homeostatic plasticity generally stabilizes a set of unsaturated weights that would be unstable under Hebbian plasticity alone (Toyoizumi et al., 2013, 2014). However, such stabilization fails if homeostatic plasticity is too slow compared to unstable Hebbian plasticity (Zenke et al., 2013). This is an example of the more general result that slow negative feedback cannot stabilize a fast, unstable positive feedback process. Some modeling studies have shown that long-term changes in synaptic weights are difficult to achieve without a “normalizing” mechanism to regulate total synaptic strength or excitability (Pérez-Otaño and Ehlers, 2005; Shepherd and Huganir, 2007; Newpher and Ehlers, 2008). The role of some chemicals as retrograde messengers in regulating presynaptic neurotransmitter release has been previously shown (Yang and Calakos, 2013; Zachariou et al., 2013; Nadim and Bucher, 2014; Padamsey and Emptage, 2014).

Diffusible messengers that have been previously implicated in activity-dependent presynaptic changes are plausible candidates also for homeostatically adjusting presynaptic release properties according to dendritic activity (Jakawich et al., 2010; Lindskog et al., 2010; Ohno-Shosaku et al., 2012).

In particular, endocannabinoids have been shown to function as retrograde messengers at CNS synapses (Castillo et al., 2012). The importance of retrograde messengers (e.g., nitric oxide, arachidonic acid, adenosine and platelet activating factor) in Hebbian plasticity and so in homeostatic processes has been proposed (Lily and Goda, 2009; Ohno-Shosaku and Kano, 2014; Wang et al., 2014). Notably, the neurotrophin BDNF, whose role in Hebbian plasticity is well established, has also been shown to play a role in homeostatic synaptic plasticity (Liu et al., 2014; Lu et al., 2014). There are research interests about the effect of a change in neurotransmitter release machinery on homeostatic presynaptic plasticity. The investigation of how homeostatic mechanisms observed at both single neuron and circuit level are integrated to regulate brain activity is a very challenging neuroscience research topic. Answering this question is potentially important if we aim to gain a comprehensive understanding on how neural plasticity in different physiological conditions is regulated to obtain high efficiency of information processing by both single and neural populations. Slow homeostatic plasticity cannot stabilize the instability effect of Hebbian plasticity. Therefore, exploring multiple regulatory pathways of interaction of these two plasticity mechanism which operate at different timescales is required to understand how they help brain to encode information (Turrigiano, 2012; Toyoizumi et al., 2014). To understand the cooperation of synaptic and non-synaptic mechanisms which operate over different timescales a model has been recently presented in which neuronal information is represented as probability distributions (Tully et al., 2014).

In this work, the main objective is to study the interaction of Hebbian plasticity and retrograde signaling which has a fast rate of diffusion from post to presynaptic sites of neurons, and influence regulation of neurotransmitter release. For this purpose, neurons with synapses that act as different information filters are simulated. The neuron's efficiency to encode its input when a different level of stimulation is presented to the neural system is measured. Hebbian mechanism and homeostatic regulation of neurotransmitter release by retrograde messenger are modeled in the synapses of 1000 neurons fully connected into a neural population where the synaptic dynamic of a single neuron of the population is studied. The model uses known basic information about biochemical interactions underlying the production of fast diffused retrograde messenger and hypothetical neurotransmitter release inhibitory machinery which is affected by pre-and postsynaptic activities. This hypothetical complex in real neurons may be composed of some protein-protein interactions or the activity of a multi-subunits protein which is activated by independent or dependent pathways. The motivation of such complex mechanisms for inhibiting neurotransmitter release is the increased evidence that supports the role of different proteins and biochemical pathways in neuronal activities. The effect of each individual mechanism and in combination with each other is studied. Moreover, the effects of integrating the homeostatic regulation of feedback inhibition by inhibitory neuron with modeled synapses on encoding efficiency of single neurons are studied. In the next sections, the details of the dynamic model and the simulations are presented. The overall importance of the results is addressed in discussion.

## Materials and methods

### Model architecture

A neuron in biological neural systems receives a large number of spikes from other neurons via synapses. These spikes are then integrated and transmitted by generating spike trains to other neurons. Such activity should be stable and efficient to transfer information. In order to simulate such complicated information processing mechanism by a single neuron, we consider a feed-forward neural layer composed of 1000 neurons fully connected to the second neural layer (100 neurons) in which we measure single neurons' encoding efficiency. Each neuron in the second layer is connected via a single synapse to neurons in the second layer. The neural activity of each of 1000 neurons is modeled as a probability of generating a spike in each time bin equal to 10 ms. The firing probability of neurons demonstrates the stimulus intensity detected by the neural system. The intensity of input to neurons may vary in different environment where the intensity of a stimulus changes over time. Therefore, it is highly vital to be able to encode fluctuating environmental stimuli by brains.

The spiking activity of single neurons was modeled using an integrate and fire model (Equation 1).

(1) $$k\frac{dV}{dt}=-g_{leak}\left(V-V_{rest}\right)+\sum I(t)$$

Where ∑ *I* (*t*) is the sum of input currents from the presynaptic neurons into postsynaptic site of a single neuron.

Table 1 shows the electrophysiological parameters used in the study (Wüstenberg et al., 2004).

**Table 1:** **Parameters of the integrate and fire neuron model used in this study**.

**Parameter**	**Value**
V_rest_ resting potential	−84 mV
V_thresh_ threshold of spiking	−25.8 mV
V_recov_ recovery threshold	−40.2 mV
V_spike_ spike potential	9.5 mV
g_leak_ membrane conductance	0.26 nS
*k* membrane capacitance	4.0 pF

### Synaptic modeling

**Figure 1:**
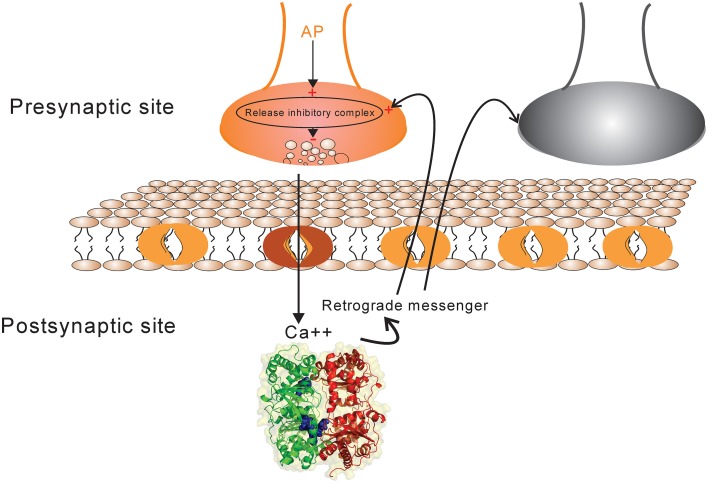
**The schematic of synapse model**. Action potential in each activated synapse (presynaptic site shown by orange) may lead to neurotransmitter release according to the initial probability of release. Modified release probability induced by the activity of release inhibitory complex can change the pattern of neurotransmitter release which induces current into the postsynaptic site. The current influx is also affected by synaptic efficacy which is determined directly by presynaptic spike trace and indirectly by Hebbian mechanism. The retrograde messenger is produced in the postsynaptic site and is released into the presynaptic site where it affects release probability complex. The sum of current influx into the postsynaptic sites triggers a single neuron to generate a spike train in response to a stimulus presentation to the neural population (presynaptic neurons). The spiking pattern of pre-and postsynaptic neurons can modify presynaptic neuron's activity and synaptic efficacy between pre-and postsynaptic neurons as well by a Hebbian mechanism.

A synapse between neurons is modeled using simplified known mechanistic events (Figure 1). When an action potential reaches the presynaptic site it leads to an increase in intercellular calcium in the presynaptic site which consequently activates some biochemical pathways by activating proteins and protein-complexes. An action potential may also lead to neurotransmitter release which causes current into the postsynaptic site. In this work, it is assumed that the current into the postsynaptic site induces the production of retrograde messenger which is diffused rapidly into the presynaptic site. Moreover, the sum of currents into the postsynaptic site triggers any single neuron to generate spikes train according to integrate and fire neuron (Equation 1). Retrograde messenger can trigger some pathways that interact with activated proteins in presynaptic neuron to activate a complex that may inhibit neurotransmitter release with different probabilities.

Hebbian synaptic plasticity is modeled in synapses such that its effect on a single neuron efficiency in combination with neurotransmitter regulation or independently is studied.

In each simulation study, a random set of neurons of 1000 neuron population is activated with a firing probability. The number of activated neurons is extracted according to Gaussian distribution with mean equal to 500 and variance equal to 50.

The current influx into the postsynaptic site is modeled as Equation (2).

(2) $$I\;\left(t\right)\;=\;\omega \;\left(\frac{t}{\tau }\right)\;e^{\frac{-t}{\tau }}\;\sum_{tp}\delta \left(t-tp\right)\;\tau \;=\;10\;ms$$

Where τ is the decay rate of current and ω is the synaptic weight between each activated single neuron in the second layer and a neuron in the first layer. ∑_*tp*_δ (*t* − *tp*) is the Dirac function.

The activity trace of spike in the presynaptic site was modeled using Equation (3).

(3) $$\dot{C}=\frac{-1}{\tau_{c}}\;\left(C+\Delta \sum_{tp}\delta \left(t-tp\right)\right)\;\tau_{c}=100\;ms$$

The change of synaptic efficacy is modeled using Equation (4).

(4) $$\dot{\omega }\;=\;Cd$$

Where *d* is the dopamine level in each time bin which is generated by high firing rate of dopaminergic neuron (equal to 0.9) (Equation 5).

(5) $$\begin{array}{l} \dot{d}\;=\;\frac{-1}{\tau_{d}}\;\left(d+\sum_{t_{d}}\delta \left(t-t_{d}\right)\right)\\ \tau_{d}=20\;ms \end{array}$$

To model Hebbian synaptic plasticity we used a simple rule shown in Table 2.

**Table 2:** **Hebbian learning rule used in the model**.

Presynaptic state	1	1	0	0
Postsynaptic state	1	0	1	0
Δ	1	−1	−1	0

Table 2 shows that when spiking of a neuron in the first layer is followed by spike in a neuron in the second layer, it may lead to higher synaptic efficacy between pair of neurons according to Equations (3) and (4).

Total produced retrograde messenger (RM) at the end of each time bin is generated according to Equation (6) such that just high levels of current can generate effective levels of retrograde messenger.

(6) $$RM\;=\;\frac{{\left(\sum I\right)}^{2}}{1+{\left(\sum I\right)}^{2}}$$

It is assumed that at the end of each time bin retrograde messenger is accumulated in the presynaptic site and its dynamics is modeled using Equation (7).

(7) $$\begin{array}{l} \dot{\text{​​}RM_{trace}}=\;\frac{-1}{\tau_{r}}\;\left(RM_{trace}\right)+RM\\ \;\tau_{r}=\;400\;ms \end{array}$$

The activity of the complex which may inhibit neurotransmitter release (*R_inh_*) is modeled as Equation (8).

(8) $$\begin{array}{l} \dot{R_{inh}}\;=\;\frac{-1}{\tau_{inh}}\;\left(R_{inh}\right)+RM_{trace}C\\ \;\tau_{inh}=\;200\;ms \end{array}$$

**Figure 2:**
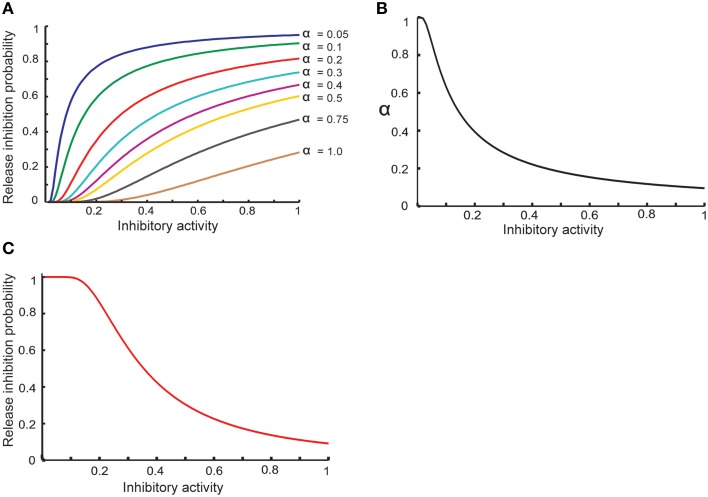
**Modeling homeostatic regulation of neurotransmitter release. (A)** The relationship between the inhibitory complex activity and release inhibition probability for different parameter values between 0.05 and 1. The plot demonstrates a non-linear relationship between inhibitory complex activity and release inhibition probability for different model parameter (α) values. Higher inhibitory complex activity needs a lower α value while low inhibitory complex activity needs a higher α value. These observations motivated to define an exponential relationship between inhibitory complex activity and α value **(B)** which lead to non-linear relationship of inhibitory complex activity and release inhibitory probability **(C)**.

Equation (8) shows that the activity of hypothetical complex to inhibit neurotransmitter release depends directly on the concentration of retrograde messenger in the presynaptic site and spike trace in the presynaptic site. To model probability of neurotransmitter release inhibition by inhibition-complex activity, we assume that this probability is changed such that higher activity can lead to higher probability (Equation 9). We assume that α should be decreased when the activity is raised (Equation 10). Hence, the probability of neurotransmitter release as a function of complex activity is presented as Equation (11) and is shown in Figure 2.

(9) $$P_{inh}\;=\;e^{\frac{-\alpha }{R_{inh}}}$$

(10) $$\alpha \;=\;1-e^{\frac{-0.1}{R_{inh}}}$$

Hence,

(11) $$P_{inh}\;=\;e^{\frac{\frac{-0.1}{\left(e^{R_{inh}-1}\right)}}{R_{inh}}}$$

(12) $$P_{rel}\;=\;\left(1-P_{inh}\right).P_{init}$$

Equation (12) shows the relationship between release probability (*P_rel_*) and inhibition probability (*P_rel_*) regarding initial neurotransmitter release probability (*P_init_*). In order to investigate the role of different kind of synapses in information processing by neurons, it is highly important to examine different initial release probability. Therefore, three initial release probabilities were considered as 0.25, 0.55, and 0.85 for synapses as high, band and low pass filters, respectively.

A challenge in modern neuroscience is how to measure the efficiency of a neural population to encode information that is received by neurons and is encoded as spiking patterns. For this purpose, information theory has proposed some measures including mutual information that can be measured by different approaches to study the role of structural and physiological parameters involved in neural systems of different senses (van Steveninck et al., 1997). Recently, a geometrical approach has been introduced that aims to measure neural system efficiency by the calculation of defined words in a given neural response (Faghihi and Moustafa, 2015). In the current study, this approach is used to measure the role of different parameters including different synapses in a single neuron's efficiency to encode its inputs. For this purpose, words composed of zeros and ones corresponding to non-spike and spike in each time bin, respectively, are defined with length equal to three. The frequency of each word in the spiking train is calculated such that any spiking train is represented as an ordered vector with length equal to eight. The stimulus intensity as firing probability of neurons in the first layer is changed 5% for probabilities from 0.5 to 0.95. For probability of firing equal to 1, the firing probability is decreased 5%. The spiking train of a single neuron in the second layer represented as vectors are used to measure the distance of neural responses as a measure how the spiking has encoded the fluctuation in its input.

## Results

Stimuli with different intensities were presented to the input layer as different firing probability of neurons in neural population (1000 neurons connected fully to second neural layer). The input layer triggers neurons in the second layer to spike with different frequency that depends on model's synaptic or network parameters. For high initial release probability of synapses between the first and second neural layer, the efficiency of a neuron of the second layer to encode stimulus information was measured for different parameter values and different assumptions about synaptic mechanisms. To model encoding efficiency of a single neuron in the second layer as a function of synaptic mechanisms and neural architectures, the inhibition of neurotransmitter release plays a critical role in this study.

Figure 2A shows the basic assumption about the relationship between release inhibitory activity in the presynaptic neuron and release inhibition probability. α value determines the dependency of release probability on level of inhibitory activity. In order to define a homeostatic regulation of release inhibition probability by inhibitory activity, the model assumes that a decrease in α value when inhibitory activity is raised. Figure 2B shows the relationship between α value and inhibitory activity in this study. Hence, the modeled relationship between inhibitory activity and release probability (Equation 11) is illustrated in Figure 2C. By homeostatic change of release probability, in different synaptic conditions and different assumptions used in the study, encoding efficiency of a single neuron was measured.

**Figure 3:**
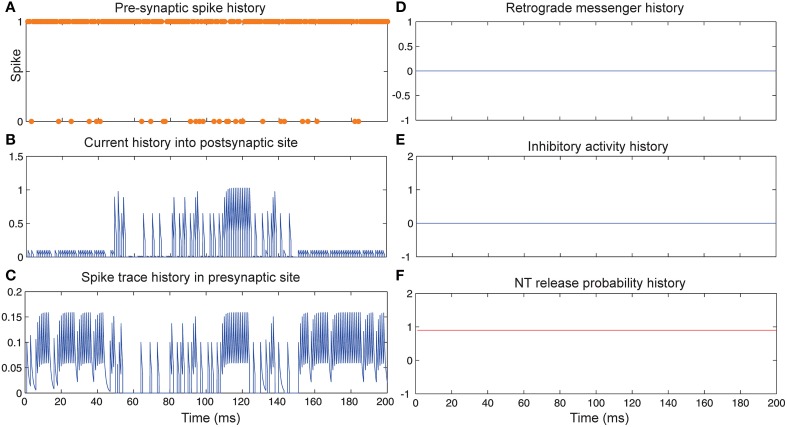
**The dynamics of model's parameters in the presence of Hebbian mechanism and absence of homeostatic regulation of neurotransmitter release. (A)** Spike train history of a presynaptic neuron with firing probability equal to 0.85 in 200 time bins. **(B)** Current history into the postsynaptic site triggered by presynaptic neuron's spiking. In time bins between 50 and 150 Hebbian rule was applied to the model while in the postsynaptic site production of retrograde messenger was blocked. **(C)** Spike trace history in the presynaptic site in time bins between 50 and 150. Spike trace activity is affected by both spiking pattern of presynaptic neuron and Hebbian mechanism. **(D–F)** No change in retrograde messenger concentration in the presynaptic site is observed. This leads to very low activity in inhibitory complex (equal to zero) and consequently in neurotransmitter release probability (equal to initial release probability).

Figure 3 shows the change of the models' parameter values of a neuron with synapses as a low pass filter (initial release probability equal to 0.85) in the presence of Hebbian mechanism without modeling homeostatic regulation of neurotransmitter release. In the absence of retrograde messenger production in the postsynaptic site, no change in neurotransmitter release is induced in the presynaptic site in the time bin between 50 and 150. In the other time bins, a single neuron spikes in the absence of both mechanisms. Figure 3A shows a presynaptic neuron spiking when a high stimulus intensity was presented to the first neural layer as firing probability equal to 0.85. This spiking pattern is used in all simulations in order to compare the parameter values in different conditions. The existence of Hebbian mechanism leads to high levels of current into the postsynaptic neuron (Figure 3B). Hebbian mechanism also induces changes in spike trace activity in the presynaptic neuron in time bins between 50 and 150 (Figure 3C). In the absence of retrograde messenger production in the postsynaptic site, no changes in release inhibitory activity in presynaptic site are observed (Figures 3D–F). These activities of neurons in the first layer result in a spiking train of neurons in the second layer. The spiking activity of a single neuron in the second layer is presented in **Figure 6B**.

**Figure 4:**
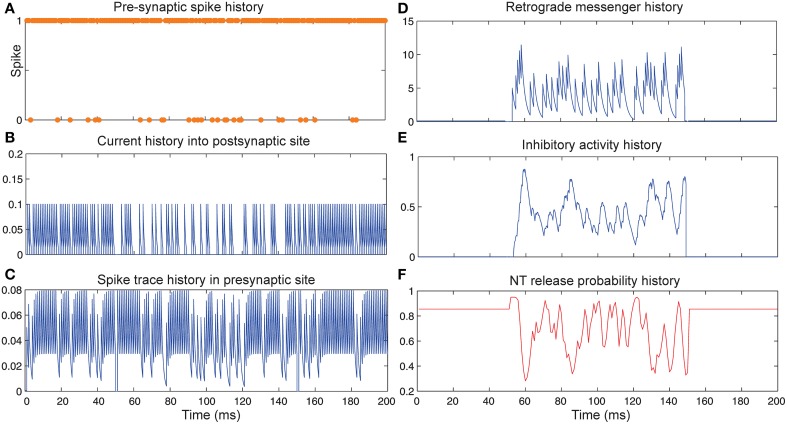
**The Dynamics of model's parameters in the presence of homeostatic regulation of neurotransmitter release and absence of Hebbian mechanism. (A)** Spike train history of a presynaptic neuron with firing probability equal to 0.85 in 200 time bins. **(B)** Current history into the postsynaptic site triggered by presynaptic neuron's spiking. In time bins between 50 and 150 homeostatic regulation of neurotransmitter release was applied by generation of retrograde messenger in the postsynaptic site and its effect in presynaptic neuron. **(C)** Spike trace history in the presynaptic site in time bins between 50 and 150. Spike trace activity is affected by both spiking pattern of the presynaptic neuron. **(D)** Retrograde messenger is generated in the postsynaptic site and received by the presynaptic site as a consequence of the presynaptic neuron activity. **(E)** Inhibitory complex activity is affected by both retrograde messenger and spike trace activity in time bins between 50 and 150. **(F)** Neurotransmitter release probability is determined by the inhibitory complex activity.

Figure 4 shows the change of model's parameter values of a neuron with synapses as low pass filter (initial release probability equal to 0.85) in the presence of homeostatic regulation of neurotransmitter release and the absence of Hebbian mechanism in time bins between 50 and 150. Retrograde messenger is produced by the postsynaptic neuron in response to the presynaptic current and is diffused into the presynaptic neuron (Figure 4D) which leads to an increase of inhibitory activity in the presynaptic neuron (Figure 4E). Consequently, it leads to changes in neurotransmitter release probability (Figure 4F). Overall activities of the presynaptic neurons induce changes in: spiking activity of the presynaptic neuron (Figure 4A), current influx into the post-synaptic neuron (Figure 4B) and spike trace induced activity (Figure 4C). The spiking activity of a single neuron in the second layer is presented in **Figure 6C**.

**Figure 5:**
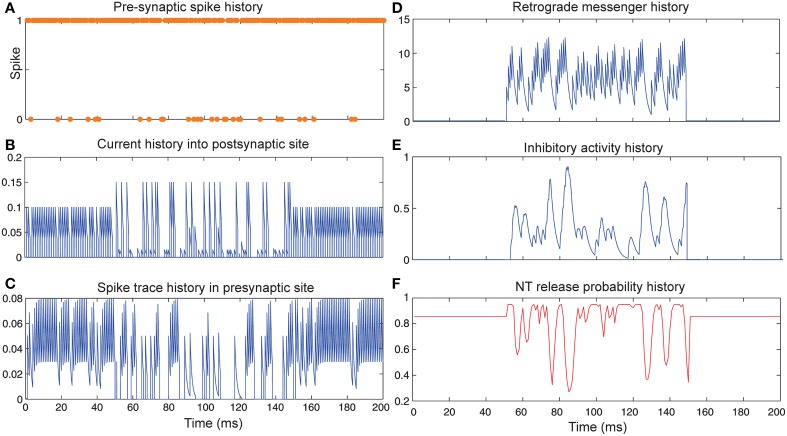
**The Dynamics of model's parameters in the presence of homeostatic regulation of neurotransmitter release and Hebbian mechanism. (A)** Spike train history of a presynaptic neuron with firing probability equal to 0.85 in 200 time bins. **(B)** Current history into the postsynaptic site triggered by the presynaptic neuron's spiking. In time bins between 50 and 150 homeostatic regulation of neurotransmitter release was applied by generation of retrograde messenger in the postsynaptic site and its effect in the presynaptic site. **(C)** Spike trace history in the presynaptic site in time bins between 50 and 150. Spike trace activity is affected by both spiking pattern of the presynaptic neuron and Hebbian mechanism. **(D)** Retrograde messenger is generated in the postsynaptic site and received by the presynaptic site as a consequence of the presynaptic neuron activity. **(E)** Inhibitory complex activity is affected by both retrograde messenger and spike trace activity in time bins between 50 and 150. **(F)** Neurotransmitter release probability is determined by inhibitory complex activity.

**Figure 6:**
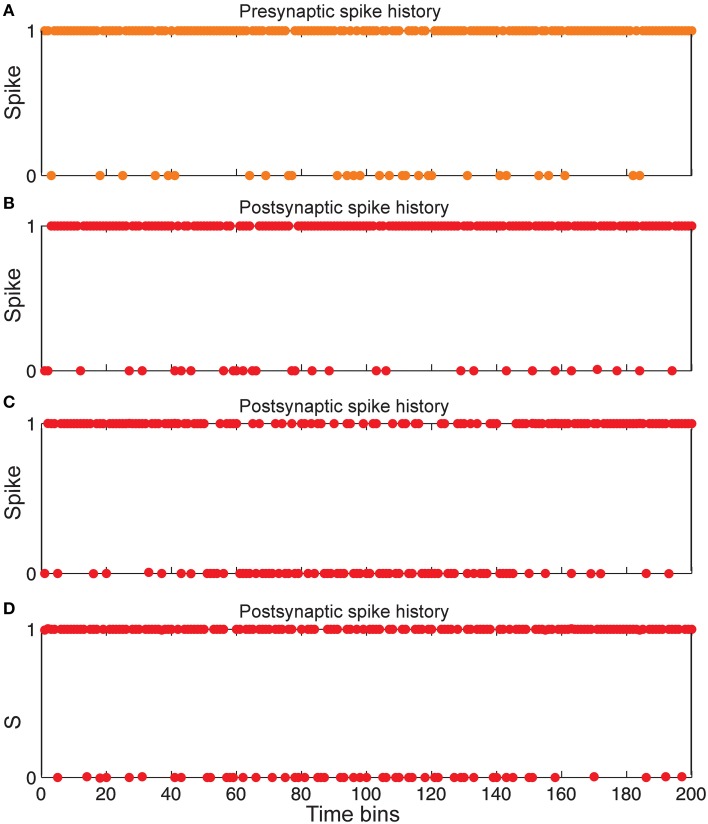
**Comparing spiking in pre-and postsynaptic neurons for different synaptic conditions. (A)** Spiking history of a presynaptic neuron in response to stimulus presentation as firing probability equal to 0.85. (**B)** Postsynaptic spiking history in the presence of Hebbian mechanism and in the absence of homeostatic regulation of neurotransmitter release induced by retrograde messenger. **(C)** Postsynaptic spiking history in the absence of Hebbian mechanism and in presence of homeostatic regulation of neurotransmitter release induced by retrograde messenger. (**D)** Postsynaptic spiking history in presence of both Hebbian mechanism and homeostatic regulation of neurotransmitter release induced by retrograde messenger.

Figure 5 shows the change of model's parameter values of a neuron with synapses as low pass filter (initial release probability equal to 0.85) in the presence of both homeostatic regulation of neurotransmitter release and Hebbian mechanism in the pre- and postsynaptic neurons. The current influx into postsynaptic neuron (Figure 5B) is affected by both mechanisms. Spike trace activity is affected by Hebbian mechanism (Figure 5C). The retrograde messenger level in the presynaptic neuron, inhibitory activity and neurotransmitter release probability is presented in Figures 5D–F, respectively. The spiking activity of a single neuron in the second layer is presented in Figure 6C.

Figure 6 shows that the spiking frequency of a single neuron in the second layer which is highly dependent on the synaptic mechanisms in the simulations. Hebbian mechanism in the absence of controlling of neurotransmitter release leads to a very high firing rate in single neurons in the second layer (Figure 6B). In the presence of inhibition of neurotransmitter release while the Hebbian mechanism was blocked, a lower firing rate of a single neuron in the second layer is observed (Figure 6C). When the model included both Hebbian and neurotransmitter release mechanisms, simulations show moderate firing rates in the spiking trains of a single neuron in the second layer (Figure 6D).

The main aim of this study is to measure efficiency of a single neuron in the second layer to encode its input. The efficiency measure used in this study allows the study of efficiency of a single neuron to encode fluctuation in their input as a vital capability of the animal brain to live in dynamic environments. For this purpose, the encoding efficiency was measured for different synaptic conditions at different firing probabilities of the input layer.

**Figure 7:**
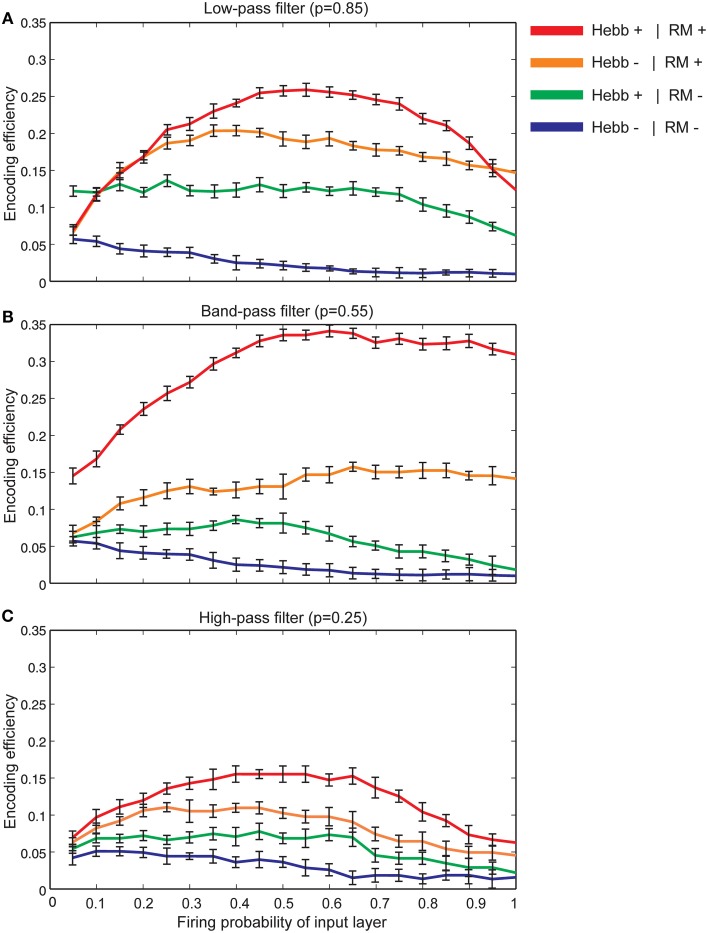
**The encoding efficiency of a single neuron in different synaptic conditions. (A)** Synapses as low-pass filters. Initial release probability was set to 0.85. The maximum efficiency was observed when both Hebbian mechanism and homeostatic regulation of neurotransmitter release were modeled (red line). Homeostatic regulation of neurotransmitter release in the absence of Hebbian mechanism leads to lower efficiency in comparison to the presence of both mechanisms in the modeled synapse (brown line). The combined presence of Hebbian mechanism and the absence of homeostatic regulation of neurotransmitter release lead to a higher efficiency in comparison to efficiency in the absence of both mechanisms (shown as green and blue lines, respectively). Hebb+ stands for existing Hebbian mechanism and RM stands for existing of retrograde messenger based induced activity. **(B)** Synapses as band-pass filters. Initial release probability was set to 0.55. In comparison to low pass-filters, when both Hebbian mechanism and homeostatic regulation mechanism of retrograde messenger exist, studied a single neuron demonstrates higher encoding efficiency. **(C)** Synapses as high-pass filters. Initial release probability was set to 0.25. In comparison to low and band-pass filters, studied single neuron shows low encoding efficiency for all synaptic conditions.

Figure 7A shows that for synapses that act as low-pass filters (initial neurotransmitter release probability equal to 0.85), the maximum efficiency of a single neuron is obtained when Hebbian mechanism and homeostatic regulation of neurotransmitter release are integrated and interact with each other in the model. However, in the absence of Hebbian mechanism or homeostatic regulation of neurotransmitter release the efficiency of a single neuron is found to be low. Minimum efficiency is obtained when synapses without both mechanisms are modeled.

Figure 7B shows the encoding efficiency of neurons with synapses which act as band-pass filters (initial neurotransmitter release probability equal to 0.55) for different synaptic mechanisms. The comparison of these results with neurons with synapses that act as low-pass filters revealed a higher encoding efficiency when both Hebbian plasticity and homeostatic regulation of neurotransmitter release exist in the synapses. When initial neurotransmitter release probability was set to 0.25 (synapses acting as high-pass filters) the encoding efficiency for all synaptic conditions is remarkably lower than low and band pass filters (Figure 7C).

**Figure 8:**
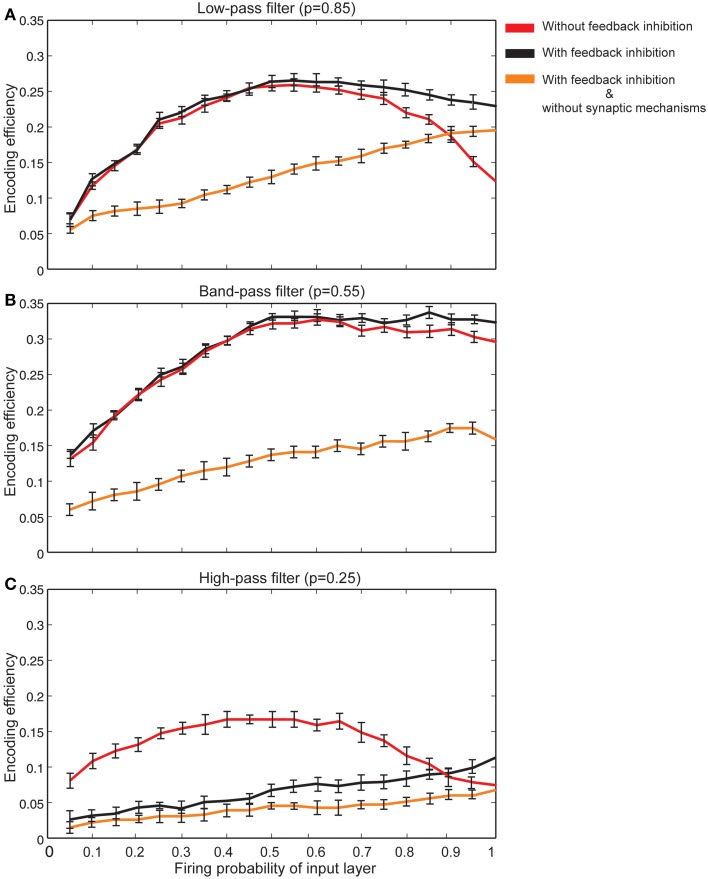
**The effect of feedback inhibition in efficiency of a single neuron for different types of synapse. (A)** Synapses as low-pass filters. Initial release probability was set to 0.85. In the presence of feedback inhibitory neuron the efficiency of a single neuron to encode stimulus presented to the neural system is higher for high firing probability of input layer (for firing probability larger than 0.6). **(B)** Synapses as band-pass filters. Initial release probability was set to 0.55. In comparison to low-pass filters, in the presence of feedback inhibitory neuron the efficiency of a single neuron to encode stimulus is higher. **(C)** Synapses as high-pass filters. Initial release probability was set to 0.25. Neuron shows low levels of encoding efficiency with or without feedback inhibitory effect.

The effect of feedback inhibition was studied in this work in combination with Hebbian mechanism and homeostatic regulation of neurotransmitter release. Figure 8A shows that for neurons with synapses acting as low pass filters (high initial release probability equal to 0.85) when high firing probability of the input layer is presented to the neural system, feedback inhibition helps a single neuron keep its efficiency at high levels. For high pass filters (low initial release probabilities set to 0.25) the association of Hebbian learning with homeostatic regulation of neurotransmitter release independently or in combination with feedback inhibition does not help neurons show high levels of encoding efficiency (Figure 8C). The highest encoding efficiency was obtained when encoding efficiency of neurons with synapses as band-pass filters were simulated (Figure 8B).

## Discussion

New explorations have shown different kinds of neuronal plasticity and neuromodulations that influence neural communication. Neuromodulators can exert effects at different timescales from short term to persistent long term regulations. The temporal dynamics of neuromodulator release plays an important role in the modulation of neural circuits, yet its effect on circuit output is not easy to understand (Marder, 2012). However, it is required to explore integrative functionalities of neuromodulators and plasticity mechanisms in network dynamics. For a better understanding of the cellular event underlying short and long term neuronal plasticity and network dynamics, a new generation of models and theories is required (Doya et al., 2002; Dayan, 2012).

In this work, an approach was applied to measure encoding efficiency which is based on counting the frequency of defined words in a spiking pattern. The method measures the efficiency of a neuron to detect fluctuation in its inputs.

The activity of a neuron may be affected by some mechanisms at network levels like feed-back and feed-forward inhibition such that any abnormality in these neurons may cause some mental disorders (Brown et al., 2015). Therefore, in this study the question that was addressed was how neuron encoding efficiency is determined in an integrated paradigm in which Hebbian learning rule and retrograde messenger effect on neurotransmitter release exist in synapses. Specifically, it is not known what potential roles played by inhibitory neurons that widely exist in neural systems in such complicated cellular events.

To address key questions related to the possible effect of the Hebbian learning rule and retrograde messengers on the presynaptic neurons and their role in homeostatic regulation of spiking activity, we developed a hypothesis that presume a molecular machinery which is responsible for inhibiting neurotransmitter release of the neuron. Such machinery may be a cellular pathway or a set of protein-protein interactions such that its activation depends on the effective presence of a spike trace (molecular changes induced by action potential) and induced effects of diffused retrograde messenger from postsynaptic neurons. The importance of dependency of release inhibition activity on spike trace is to prevent any non-specific activation of molecular machinery by diffused retrograde messenger to non-activated synapses in neural networks. Our modeling and simulation results suggest novel experiments to explore such molecular machinery or biochemical pathways. In this model, spiking of the presynaptic neurons (if associated with neurotransmitter release) may trigger postsynaptic neurons to produce locally retrograde messenger which is rapidly received by the presynaptic neuron. Such assumption suggests gaseous chemical like nitric oxide as a retrograde messenger candidate for this hypothesis (Hardingham et al., 2013; Neitz et al., 2014; Sagi et al., 2014). Non-gaseous chemicals may have a longer time scale to affect presynaptic neurons so their contribution in interaction with Hebbian mechanism may lead to different results. If associated with effective levels of spike trace, received retrograde messengers received by presynaptic neurons may lead to the inhibition of neurotransmitter release. A decrease in synaptic weight is obtained if the postsynaptic neuron spikes in response to the sum of its input (according to the Hebbian rule, in Table 2). In this study, we modeled synapses in which Hebbian plasticity and neuromodulatory mechanism as fast diffused outward of retrograde signaling exist and interact in short term timescales. The best candidate for such retrograde signaling is nitric oxide which is produced and diffused by the stimulation of the sum of input current from presynaptic sites. Such assumption gives rise to the existence of a loop between pre-and postsynaptic sites as follows: retrograde messenger from postsynaptic site modifies release probability of neurotransmitter. The released neurotransmitter in combination with synaptic efficacy determines the current influx into postsynaptic site. The spike timing of postsynaptic activity triggered by total current affects the Hebbian plasticity mechanism. Consequently, synaptic efficacy between pre-and postsynaptic sites is changed and so it leads to a change in current into postsynaptic site which modify retrograde messenger production in the next time bins. Hence, the combination of both mechanisms at the network level has resulted in a firing rate of postsynaptic neuron (single neuron) at moderate level (not too high or too low) when high stimulus intensity was presented to the neural system. In such stimulus presentation conditions, in the absence of retrograde messenger effect on presynaptic neuron, due to Hebbian learning rule, postsynaptic neurons generate spiking with high frequency which leads to a low encoding efficiency. Retrograde signaling in the absence of Hebbian learning rule can help neurons to control their spiking activities, but encoding efficiency does not reach high levels because release inhibitory activity is not strong enough to control spiking rate. Moreover, adding the simulation of effective inhibitory feedback by an inhibitory neuron on the network shows its vital role in encoding efficiency of single neurons when combined with Hebbian mechanism and retrograde messenger comparing to the efficiency in the absence of these synaptic mechanisms (Faghihi and Moustafa, 2015). These simulations assign a critical role for nitric oxide as a known retrograde messenger with desired properties for the proposed hypothesis. Therefore, our simulation studies provide new predictions and additional experiments on the role of this chemical in the nervous system.

Retrograde messenger with different timescales of operation may play other roles in homeostatic regulation of neuronal spiking stability. One may be its role as an error signal. Such error signal as the difference between the basal level of retrograde messenger or synaptic efficacy and updated level can act as a correction mechanism to stabilize synaptic activity (Davis, 2006). Therefore, the correction mechanism of different retrograde messengers in combination with Hebbian plasticity and homeostatic plasticity should be considered in future computational modeling work.

In our simulations, a high firing rate was used for dopaminergic neurons in order to keep dopamine at constant levels such that the dynamics of synaptic efficacy is affected only by changes in the spike trace and Hebbian learning rule. In future work, one may examine different levels of dopaminergic neurons' firing rate and different learning strategies to study its effect on network activity when assumptions about retrograde messenger's effect or feedback inhibition are either changed or fixed. It is known that synapses may vary in their molecular compositions which lead to demonstrate a different initial release probability (Fernandez-Chacon et al., 2001; Kavalali, 2015). Accordingly, it is important to consider synapses with different initial release probability and its effect on encoding efficiency. This theoretical study assigns a critical role for homeostatic regulation of neurotransmitter release by fast diffused retrograde messenger and Hebbian plasticity in efficient neuronal encoding tasks when synapses are acting as low or band pass filters. Moreover, the model predicts that there are other synaptic mechanisms for neurons with synapses which act as high-pass filters that enable them to encode their inputs with different levels of intensity.

Modeling work in the current study is based on a simple mechanistic implementation of complicated molecular events in which some biophysical properties of agents were simplified. For example, we did not model diffusion of retrograde messenger. Assuming that it acts at very low distances, it can affect partially activated synapses in the network. Our understanding of the cellular mechanism of neurotransmitter release machinery and its inhibition mechanisms, especially the time scale of different underlying mechanisms, may lead to a modification of the dynamics of the model and basic assumptions. However, such modification and improvements need many experiments on the hypothesis and its mechanistic details. Another possibility to improve the model is to consider how structural plasticity (the dynamic connectivity of neurons over time) interact with the change of information flow into a neuron (Lamprecht and LeDoux, 2004; Yin and Yuan, 2015).

The simulations presented in this study predict that impairment in any part of such complex cellular mechanisms may lead to a deficiency in neural encoding of neurons and neural populations. Therefore, it offers an explanation of the role of genetic mutations that may affect biochemical pathways of information processing in neurons which gives rise to synaptic diseases (Chakroborty et al., 2012; Grant, 2012).

Modeling and simulation studies in combination with experiments can help improve our understanding of neural encoding and decoding in different physiological conditions, specifically where cellular parameters like biophysical properties of channels or changing biochemical pathways are not easily assessable to be modified by experimental techniques. The importance of such efforts are better realized when we consider the role of theoretical studies in measuring quantities that are not basically determined by experiments at molecular, cellular or network levels. One of the best examples of such quantities is to measure information transferred by neurons. Until now mainly information theory and some mathematical measures have been used for this purpose. Therefore, applying such measures in simulations especially if they are based on information about cellular architecture and functionality can play a very critical role in designing research plans to explore basic principles of information processing in animal and human the brain.

### Conflict of interest statement

The authors declare that the research was conducted in the absence of any commercial or financial relationships that could be construed as a potential conflict of interest.
